# Editorial: Health promoting settings in the 21st century: new approaches and competencies to address complexity and inequity in an increasingly globalized world

**DOI:** 10.3389/fpubh.2024.1429788

**Published:** 2024-05-21

**Authors:** Peter Delobelle, Nastaran Keshavarz Mohammadi, Irma Brito

**Affiliations:** ^1^Chronic Disease Initiative for Africa, University of Cape Town, Cape Town, South Africa; ^2^Department of Public Health, Vrije Universiteit Brussel, Brussels, Belgium; ^3^Department of Public Health, Shahid Beheshti University of Medical Sciences, Tehran, Iran; ^4^Health Sciences Research Unit, Nursing School of Coimbra, Coimbra, Portugal

**Keywords:** health promotion, settings approach, competencies, complexity, inequity

The settings approach to health was initiated by WHO following the Ottawa Charter for Health Promotion and aimed to contribute to the implementation of its holistic concept in diverse settings. The movement started with Healthy Cities and was soon followed by health promoting schools, workplaces, hospitals and healthcare services, prisons, playgrounds, neighborhoods and other new emerging settings (1). Since then, settings-based health promotion programs, research and policies have continued to grow (2) and at the onset of the twenty-first century, a consensus was reached about the complexity of settings and how to introduce and sustain health promoting changes in settings (3).

The collection of articles in this Research Topic aims to collect and highlight new approaches and competencies to address complexity and inequity in an increasingly globalized world focusing on new developments in the settings approach and innovative approaches and ways of working and thinking in settings-based policy, practice, and theory. The articles describe a range of initiatives in different settings, from remote Australian communities through university campuses to prisons and sports federations, and methods and approaches, including individual and community-based strategies, guided by reflective practice and conceptualization of new frameworks for action.

For example, McRae et al. described a qualitative process evaluation of a *community*-led health promotion initiative set during the COVID-19 pandemic in 2020. The initiative involved producing a hip-hop music video (‘HipHop2SToP') with youth in the Kimberly Region of Western Australia and showed that community-led and culturally appropriate initiatives could strengthen community ownership and create new ways of maintaining relationships with remote Aboriginal communities. Likewise, Khazaee-Pool et al. in their research illustrate how social innovation in health and community-driven engagement acted as key strategy to address COVID-19 challenges in the multicultural society of Iran by increasing equity in health services access, especially among vulnerable groups and minorities.

In *universities*, Weaver et al. demonstrated how reflective structured dialogues can be used as a tool for addressing wicked public health problems, including polarization-related attitudes among university students on controversial divisive topics such as COVID-19, mental health, and racism. They showed that participation in conversations was strongly associated with improved attitudes related to openness, tribal identity, and moral disdain. Suarez-Reyes and Van den Broucke, in their global survey of participation of university community members in Health Promoting Universities, indicated that students were more involved in information delivery, the lowest level of participation, while professors were relatively more involved in consultation strategies and design, planning and decision-making, leaving room for improvement in terms of community participation. Doré et al. in their article on promoting the interruption of sedentary behavior among university students during online classes explored the use of videos with different message strategies as a tool for behavior change, although further research on effective communication and message strategies is needed.

In *prisons*, Tesler et al. report results from a cross-sectional study among Israeli inmates, showing that most participants failed to meet recommended physical activity levels and with half reporting that their physical activity levels decreased since being in prison. Participation in health promoting activities was associated with higher levels of activity and subjective health status and significantly higher among younger males, showing that health promotion activities may play an important role in addressing the challenges of maintaining inmate health.

The potential for organized sports to promote health was highlighted by Van Hoye et al. who in their article provide guidelines to support national *sports federations* to invest in health promotion. They elaborated how the settings-based approach to health promotion can be adapted to national sports federations by clarifying theoretical concepts, providing practical applications of potential interventions based on case studies, and guidelines and tools useful for implementation.

Jenkins et al. in their conceptual review of settings for the development of health literacy pointed to the need to identify and conceptualize *non-traditional and emerging settings* in the twenty-first century. They developed a conceptual model using a public library to propose four equity-focused antecedents to develop health literacy and located this within a “*super settings”* approach, where multiple settings work in synergy with each other.

Tong et al. in their article on developing a health literacy environment scale for Chinese *hospitals*, pointed to the complexities of an environment traditionally used to promote health literacy. They describe a process of rigorous scale development and validation resulting in a psychometrically sound instrument that provides a patient perspective for evaluating the environment which makes it easier for patients to access, understand, and use health information. Scale validation was also the subject of the article by Lynn Ho et al. who assessed the family health climate in a multi-ethnic Asian population and adapted an instrument validated in Western populations for Singapore whilst accounting for language and cultural differences. The results suggest a good psychometric profile, including after the development of shorter versions of the scales.

COVID-19 also impacted the *digital environment*, with attention to digital health literacy and online teaching. Getachew et al. in their article on the digitalization of health during COVID-19, elaborate on the role of digital support for the prevention, diagnosis, and care at individual level, and data management, outbreak tracking and pandemic surveillance at population level. Questions were raised about its cost, compatibility with existing systems, disruption in patient-provider interactions and sustainability, in turn calling for more evidence on clinical utility and economic evaluation.

The COVID-19 pandemic also showed how *primary care* plays a central role in promoting health and preventing disease, even during health emergencies. Milani et al. in their perspective showed how in Italy local primary care centers called “Houses of Community” were used as a new model of care and the nearest access point to provide continuity of care and health and social integration. They pointed to the need for multiprofessional collaborative practices between services of care, local health districts and researchers, using participative approaches in research and action, and educational programs. Lev and Ron highlighted inequities in access to health care during the COVID pandemic, demonstrating the need not only to prevent the onset and progression of chronic non-communicable diseases and to promote healthy lifestyles, but also to prepare for new infectious diseases and their long-term effects on physical and mental health. In their perspective they argue for a broad-based approach to health promotion, prevention and preparedness (HPPP) at national and global level to strengthen government commitments to the Sustainable Development Goals.

Policies to curtail environmental determinants of unhealthy behavior were discussed in the article by Alebshehy et al. in a scoping review of policies regulating the *retail environment* to reduce tobacco availability. Through a search of WHO FCTC and COP decisions, scientific and gray literature, they identified measures to regulate the retail environment, the adoption of which in WHO FCTC related decisions would probably increase their implementation worldwide.

Li et al. in a methods article on contextual and environmental factors that influence health, report a within-subjects field experiment protocol focusing on the *street environment* as a routine setting for daily activities that integrated instantaneous assessments of the environment, physical activity and health outcomes. Using state-of-the-art environmental monitoring and biosensing techniques and focusing on physically active road users, an experiment was successfully executed in Texas, showing its feasibility of capturing health effects of physical activity in various urban environments by combining environmental, behavioral and physiological sensing.

Costa-Clemens et al. in their brief research report, describe a rapid site readiness project in Latin America to test clinical trial capacity building for COVID-19 vaccine and drug development, which underscores the need for the availability of sufficient and well-trained *clinical trial sites*, especially in low- and middle-income countries.

Finally, Pescud et al. argued that while strengthening settings for prevention of chronic diseases is essential, leadership for systems change is crucial. In their article, they examined and described researcher practices for enhancing impact in prevention research using case studies. The results indicate that persuasive communication, compassion and deep listening, reflective practice, and research embeddedness can help create change within systems.

What all these studies show is that health promotion needs to be designed according to the needs and resources of the target population (group or community) and make use of nudging strategies, policy reinforcement and the reorganization of health and social action services. They show that there is investment in new approaches to increase competencies in order to address complexity and inequity worldwide. Health promotion and preparedness good practices are crucial to address the daily needs of groups and communities facing inequality, and even more so when catastrophic situations occur that affect entire populations and accentuate these conditions (4). Building health promotion competencies is essential and should be part of any training program aimed at building a skilled and competent public sector workforce (5, 6) and empowering university communities to lead this effort could bring countless benefits and return to society the investment made in training skilled technicians, thereby helping to achieve the sustainable development goals (4).

The collection of articles presented here also provide key insights into new approaches to address the different action areas of settings-based health promotion, ranging from individual to population level targets and from policy to practice, guided by sound research and reflection. The articles highlight the unwavering commitment of researchers and scholars to think and rethink twenty-first century health promotion, using the advances of digital technology, research and theoretical frameworks, focusing on equity in access to care and on pandemic prevention and preparedness, set against the backdrop of the COVID-19 pandemic. Lessons can be learnt from new settings-based models and programs, and we hope that the articles in this Research Topic will bring some food for thought and inspiration and that you will enjoy them as much as we did.

## Author contributions

PD: Conceptualization, Writing – original draft, Writing – review & editing. NK: Writing – review & editing. IB: Writing – review & editing.
